# 562. Maternal RSV Vaccine and Nirsevimab Effectiveness against Medically Attended RSV Disease in Infants, United States

**DOI:** 10.1093/ofid/ofaf695.171

**Published:** 2026-01-11

**Authors:** Fatimah S Dawood, Ayzsa Tannis, Leah Goldstein, Natasha B Halasa, Julie A Boom, Geoffrey A Weinberg, Mary A Staat, Eileen J Klein, John Williams, Jennifer E Schuster, Ariana Toepfer, Casey M Kalman, Abigail L Salthouse, Leila C Sahni, Laura S Stewart, Marian G Michaels, Daniel C Payne, Peter G Szilagyi, Rangaraj Selvarangan, Janet A Englund, Heidi L Moline

**Affiliations:** CDC, Atlanta, Georgia; Centers for Disease Control and Prevention, Atlanta, Georgia; Centers for Disease Control and Prevention, Atlanta, Georgia; Vanderbilt University Medical Center, Nashville, TN; Baylor College of Medicine, Houston, Texas; University of Rochester Sch Med & Dent, Rochester, New York; Cincinnati Children's Hospital Medical Center, Park Hills, Kentucky; Seattle Children's Hospital and University of Washington School of Medicine, Seatte, Washington; University of Wisconsin, Madison, Wisconsin; Children's Mercy Kansas City, Kansas City, MO; Centers for Disease Control and Prevention, Atlanta, Georgia; Centers for Disease Control and Prevention, Atlanta, Georgia; Centers for Disease Control and Prevention, Atlanta, Georgia; Baylor College of Medicine and Texas Children's Hospital, Houston, Texas; Vanderbilt University School of Medicine, Nashville, Tennessee; University of Pittsburgh/ CHP, Pittsburgh, Pennsylvania; Cincinnati Children's Hospital Medical Center, Park Hills, Kentucky; UCLA, Los Angeles, California; Children’s Mercy Hospital, Kansas City, Missouri; Seattle Children’s Hospital/Univ. Washington, Seattle, Washington; US-CDC, Atlanta, Georgia

## Abstract

**Background:**

During the 2023-2024 respiratory syncytial virus (RSV) season, two new RSV prevention products were recommended to protect US infants in their first RSV season, maternal RSV vaccine and nirsevimab. Using data from a 7-site respiratory virus surveillance network, we examined effectiveness of these products against medically attended RSV.

**Methods:**

Infants with outpatient, emergency department, and inpatient visits for acute respiratory illness were enrolled during the 2023-24 and 2024-25 RSV seasons. All children had molecular testing for RSV and clinical and demographic data collected from caregiver interviews, medical records, and immunization registries. A test-negative design was used to estimate maternal RSV vaccine effectiveness (VE) in children < 6 months in both seasons and nirsevimab effectiveness in infants in their first RSV season in 2024-25. Cases and controls were infants who tested positive and negative for RSV, respectively. Maternal RSV vaccination was receipt >14 days before infant birth. Nirsevimab receipt was administration >7 days before symptom onset.

**Results:**

Among 1248 infants < 6 months without nirsevimab receipt, 27/344 (8%) case and 144/895 (16%) control patients had maternal RSV vaccination; median infant age was 44 days (IQR 23-84). Maternal RSV VE was 63% (95% CI 40-77%) against medically attended RSV and 74% (95% CI 54-86%) against RSV hospitalization. Among 1362 infants in their first RSV season without maternal RSV vaccination, 60/452 (13%) case and 372/910 (41%) control patients received nirsevimab. Nirsevimab effectiveness was 78% (95% CI 69-84%) against medically attended RSV (median interval from receipt=62 days, IQR 35-96) and 82% (95% CI 72-89%) against RSV hospitalization; effectiveness was similar by RSV type and prematurity status. Nirsevimab effectiveness varied by time since receipt from 92% (95% CI 80-97%) in infants < 30 days from receipt (median interval=21 days, IQR 15-24) to 69% (95% CI 48-82%) in infants 90-164 days from receipt (median interval=111 days, IQR 102-126).

**Conclusion:**

Under real-world conditions, both maternal RSV vaccine and nirsevimab effectively prevented medically attended RSV among infants in their first RSV season, with evidence of sustained nirsevimab protection through at least 5 months from receipt.

**Disclosures:**

All Authors: No reported disclosures

